# Tibial Eminence Avulsion in a Tibial Plateau Fracture – Our Approach: A Clinical Case

**DOI:** 10.1055/s-0041-1726067

**Published:** 2021-04-19

**Authors:** Rómulo Silva, José Barreto, Filomena Ferreira, Margarida Areias, Carolina Oliveira, Bruno Alpoim

**Affiliations:** 1 Departamento de Ortopedia e Traumatologia, Unidade Local de Saúde do Alto Minho (ULSAM), Viana do Castelo, Portugal; 2 Departamento de Medicina Física e Reabilitação, Centro Hospitalar Entre Douro e Vouga (CHEDV), Santa Maria da Feira, Portugal

**Keywords:** knee, trauma, tibial fractures, arthroscopy, fracture fixation, internal

## Abstract

A middle-aged female patient with a tibial plateau fracture combined with an avulsion of the tibial eminence was treated with a combination of medial plate fixation for the plateau and an arthroscopic aided nonabsorbable suture of the eminence.

Our technique for tibial eminence avulsion fractures has no interference with tibial plateau osteosynthesis materials and has proven, once again, to have good results in the treatment of combined and complex injuries of the knee.

## Introduction

The avulsion of the tibial eminence is one of the most common injuries associated with the tibial plateau fractures. 1 2 3 4 The literature regarding this association is scarce. 5 This combination results on a complex injury pattern that demands a more elaborate approach. 1 2

Regarding fracture patterns, higher Schatzker injury types (V and VI) are more frequently found in this type of lesion. 3 There has been a major uprising of arthroscopic reduction and internal fixation (ARIF) in the treatment of persistently displaced injuries 2 3 and the importance of arthroscopy in the approach of intra-articular lesions is well documented. 2 6 7 8 9 10

We present a clinical case with a combination of a medial tibial plateau fracture with an avulsion of the tibial eminence, where we expose the approach of our institution for this type of injuries.

## Case Report

**Fig. 1:**
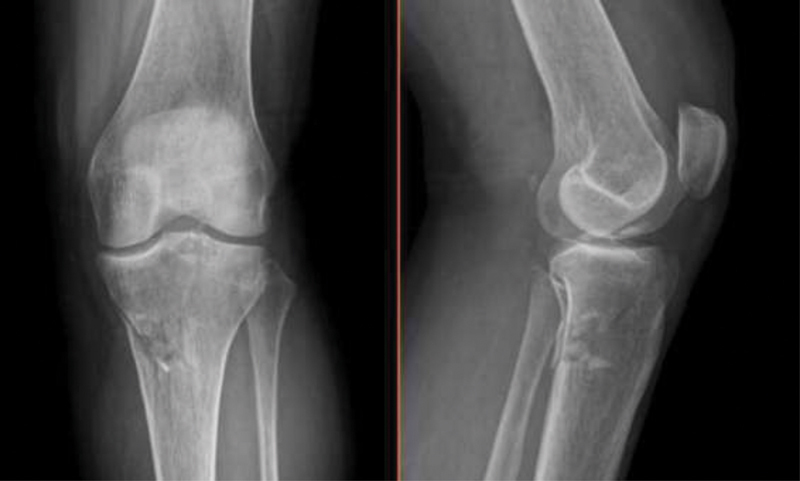
Anteroposterior and lateral radiograph in the emergency room.

**Fig. 2:**
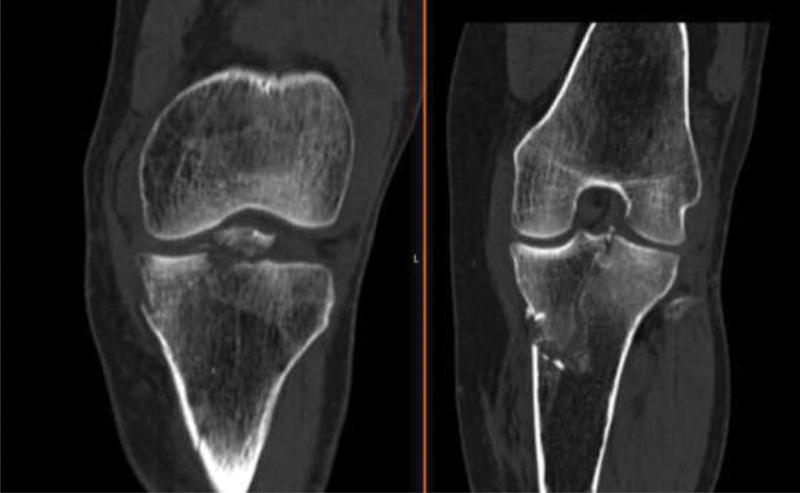
Coronal cuts of the knee computed tomography scan.

**Fig. 3:**
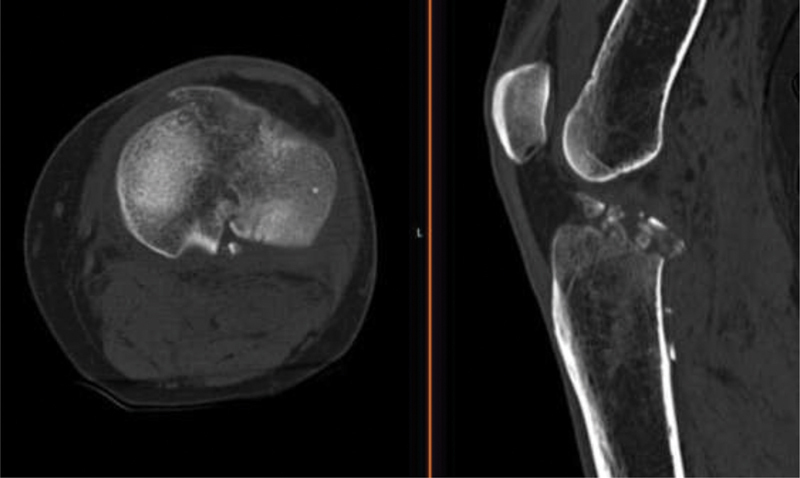
Axial and sagittal cuts of the knee computed tomography scan.

A 52 year-old female with a history of dyslipidemia suffered a fall from a chair with resultant knee trauma. She presented to the emergency room with significant knee effusion and pain, no major deformity and no neurovascular deficit. We performed an imagiological survey with radiographs ( Fig. 1 ) and CT scan ( Fig. 2 and 3 ), which showed a split fracture of the medial plateau through the tibial eminence, with dislocation and fragmentation of the eminence. The CT scan also reported a small fracture of the posterior margin of the external plateau. The patient was immobilized with a posterior splint and was prepared for the operating room.

**Fig. 4:**
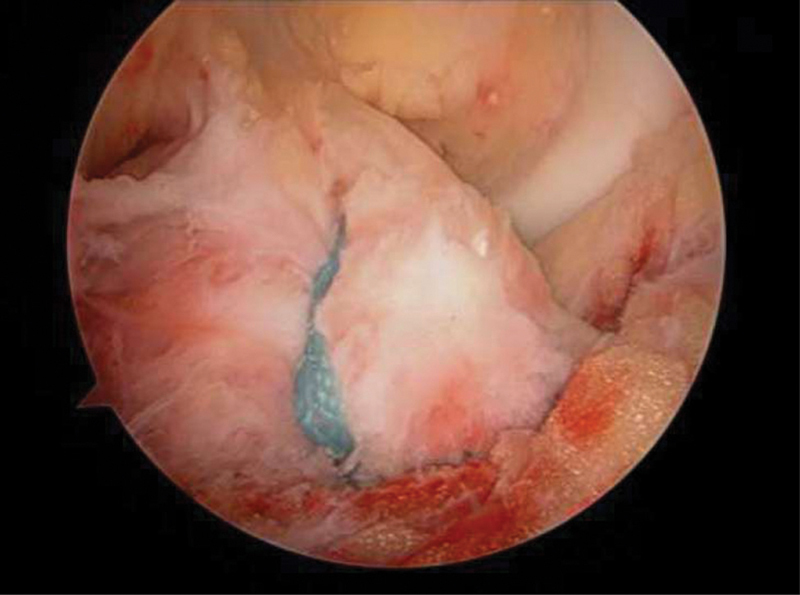
Arthroscopic view of the tape transfixing the anterior cruciate ligament and pulling down the eminence fracture.

**Fig. 5:**
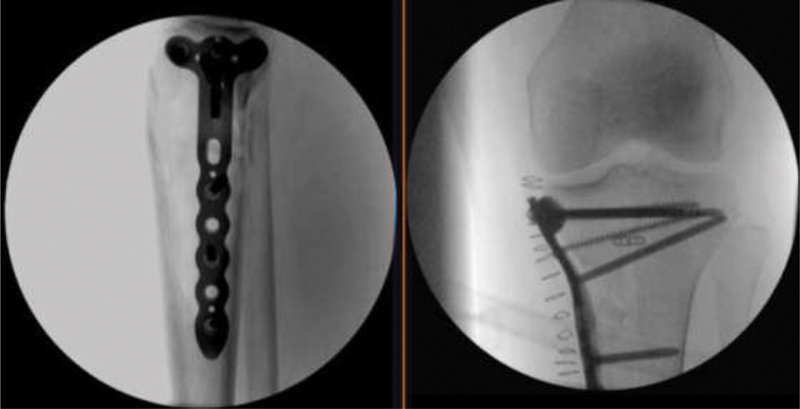
Intraoperative anteroposterior and lateral fluoroscopy views.

First, we approached the medial tibial plateau with a posteromedial incision, reduced the split fracture with help from fluoroscopy and temporarily placed the medial plate with Kirschner wires. We then advanced to a two-portal arthroscopic procedure, with removal of debris and lavage, followed by exploration of the tibial plateau fracture, and searched for associated intra-articular lesions. When satisfied with the reduction of the medial plateau, we proceeded to permanently fixate the plate in place. After this step, we went on to the tibial eminence: Since the debridement was already done and no entrapment of the meniscus was observed, we placed the ACUFEX Director Drill Guide (Smith and Nephew, Watford, England, UK), from the cruciate ligaments instrumental, in the anterior aspect of the eminence's fracture bed, and created a single tunnel through the anterior border of the proximal tibia. The next step was to pass an ULTRATAPE Suture (Smith and Nephew, Watford, England, UK) by the arthroscopic portals, then to transfix the fibers of the anterior cruciate ligament as closely as possible to the inferior bony fragment ( Fig. 4 ); then it was inserted through the tibial tunnel, reducing the eminence fracture, and fixated via an endobutton with the knee in full extension ( Fig. 5 ).

Postoperatively, the patient was placed in a De Puy (generic 0° knee imobilizing) brace with no weight bearing for 15 days. The rehabilitation program was initiated immediately postsurgery in an outpatient setting with neuromuscular electrical stimulation of the quadriceps and hamstrings with the knee in full extension. The following stage of the rehabilitation program comprehended isometric strengthening of the hip adductors and abductors, gradual removal of the brace, and passive knee mobilization with progressive degrees of flexion, with the main goal being the improvement of range of motion (ROM). At 1 month postsurgery, pain-free weight bearing was allowed, and the focus was muscular strengthening of the quadriceps, hamstrings, and gastrocnemius, starting with isometric exercises and progressing at 6 weeks postsurgery to dynamic and plyometric exercises with resisted flexion allowed in full range of motion and resisted extension at between 30 and 90° of flexion. Resisted extension at between 0 and 30° was allowed at 3 months postsurgery. Open kinetic chain proprioceptive exercises were initiated at 6 weeks, with progression to closed kinetic chain exercises.

**Fig. 6:**
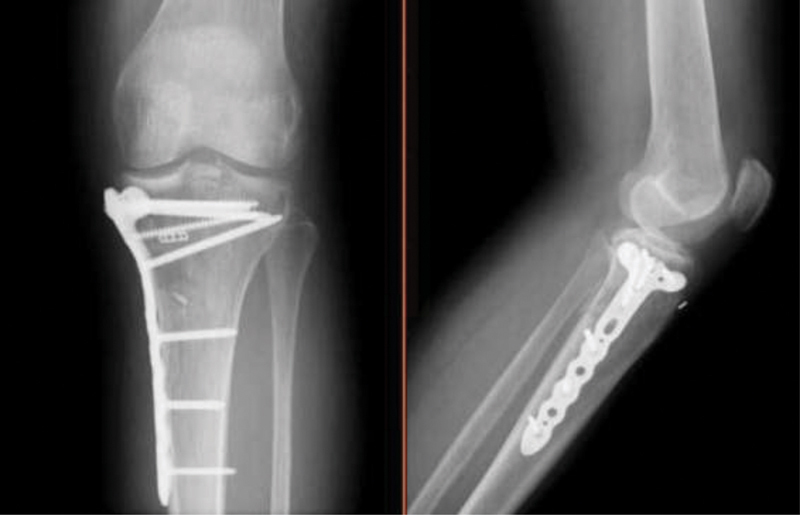
Anteroposterior and lateral radiograph at the 3-month follow-up.

**Fig. 7:**
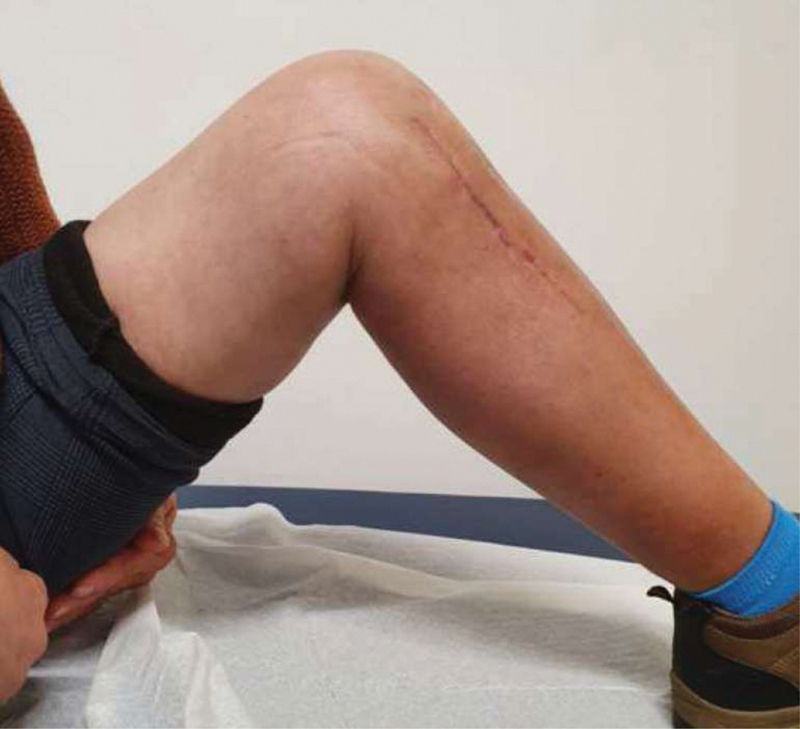
Third month post-operative flexion.

**Fig. 8:**
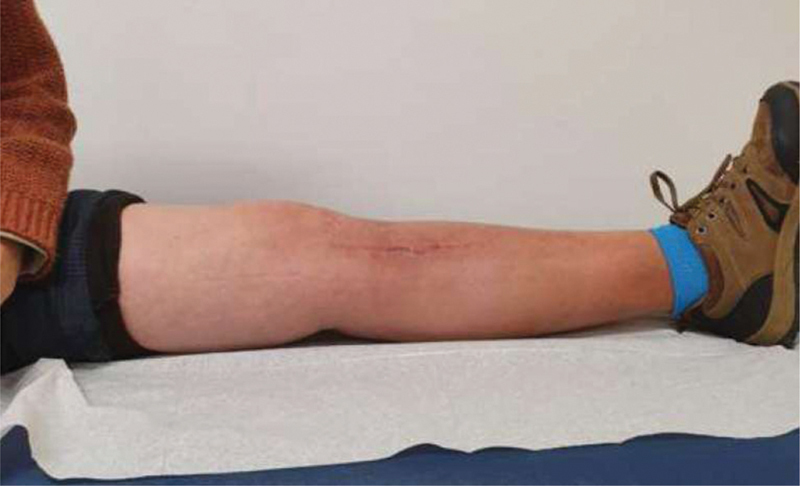
Third month post-operative extension.

At 3 months, the patient presented no pain, good radiographic alignment ( Fig. 6 ), full weight bearing, knee ROM between 0 and 90° and no crutches ( Figs. 7 and 8 ).

In the 1-year follow-up, the patient reported no limitation on her activities of daily living, with an IDKC score of 74.7, negative anterior Lachman and Drawer tests, and no impediment to everyday activities.

## Discussion

The relevance of tibial eminence fracture in the context of a tibial plateau fracture is poorly identified in the current literature, 5 but information suggests that 19.4% of tibial plateau fractures are associated with a tibial eminence fracture, and that 84.3% of the tibial spine fractures in adults coexist with a tibial plateau fracture. 2

Regarding this specific combination of injuries, we found in the literature some reports on the approaches and outcomes.

Di Caprio et al. 2 performed a study with 29 patients with fracture of the tibial plateau and of the tibial eminence and described 2 arthroscopic techniques, similar to our own, to approach these injuries, with good results. As advantages of these arthroscopic and pull-out suture techniques, they pointed out the simplicity, low rate of intraoperative complications, and the ability to properly tension the anterior cruciate ligament. This study had the particularity that all the tibial plateau fractures were treated with percutaneous screw fixation.

Huang et al. 1 also studied this combined injury in a retrospective study with a minimal follow up of 5 years, using an identical 2 tunnel technique, with results comparable to those of Di Caprio, 2 but also stating that the technique did not interfere with the plate and screw fixation of the tibial plateau fracture, which is in line with our case.

Lubowitz et al. 3 describe another equivalent technique for fixation of the tibial eminence with nonabsorbable sutures, reporting advantages of eliminating the risk of comminution of the fracture fragment, need for hardware removal and posterior neurovascular injury.

Regarding the outcomes, Konda et al., 5 when comparing patients with this combined injury with those with tibial plateau fractures alone, found slower recovery rates in the first group, but comparable functional outcomes at the 1-year follow up. Sapre et al. 11 also found good results with anatomic reduction with various arthroscopic techniques.

In the present article, we describe yet another combined surgical technique involving arthroscopy, nonabsorbable suture and plate fixation of this combined injury, with a good result at the 1-year follow-up. A timely and tailor-made rehabilitation program is also crucial to the clinical outcome. This data supports the aforementioned studies as an excellent surgical time approach to a complex lesion of the knee, with no incompatibility between the material for the tibial plateau and the fixation method for the eminence, good recovery times, and satisfiability.
