# Supplementary material for: Tibial Eminence Avulsion in a Tibial Plateau Fracture – Our Approach: A Clinical Case
Source: Rev Bras Ortop (Sao Paulo). 2021 Apr 19;59(2):e318–22. doi: 10.1055/s-0041-1726067 (PMC11006521; doi:10.1055/s-0041-1726067)
Supplement: Related texts

# Research article: Avulsão da eminência tibial em uma fratura do platô tibial – Nossa abordagem: Um caso clínico

**Keywords:** joelho, trauma, fraturas da tíbia, artroscopia, fixação interna de fraturas

## Abstract

Uma paciente de meia-idade com fratura do platô tibial e avulsão da eminência tibial foi tratada com uma combinação de fixação do platô com placa medial e sutura da eminência com fio não absorvível auxiliada por artroscopia.

Nossa técnica para fraturas com avulsão da eminência tibial não interfere nos materiais de osteossíntese do platô tibial e, mais uma vez, teve bons resultados no tratamento de lesões combinadas e complexas do joelho.

## Introdução

A avulsão da eminência tibial é uma das lesões mais comuns nas fraturas do platô tibial. 1 2 3 4 A literatura a respeito dessa associação é escassa. 5 Essa combinação gera um padrão complexo de lesão que exige uma abordagem mais elaborada. 1 2

Em relação aos padrões de fratura, os tipos mais elevados de Schatzker (V e VI) são mais frequentes neste tipo de lesão. 3 Houve um grande aumento na realização de redução artroscópica e fixação interna (ARIF, na sigla em inglês) no tratamento das lesões com deslocamento persistente 2 3 e a importância da artroscopia na abordagem das lesões intra-articulares está bem documentada. 2 6 7 8 9 10

Apresentamos um caso clínico com combinação de fratura do platô tibial medial e avulsão da eminência tibial, onde expomos a abordagem de nossa instituição para este tipo de lesões.

## Relato de Caso

Uma mulher de 52 anos com histórico de dislipidemia caiu de uma cadeira e sofreu um traumatismo no joelho. No pronto-socorro, apresentava derrame sinovial e dor significativos no joelho, sem deformidade importante ou déficit neurovascular. As radiografias ( Fig. 1 ) e a tomografia computadorizada (TC) ( Fig. 2 e 3 ) mostraram uma fratura de separação do platô medial através da eminência tibial, que apresentava deslocamento e fragmentação. A TC também revelou uma pequena fratura da margem posterior do platô externo. A paciente foi imobilizada com tala posterior e preparada para a cirurgia.

**Fig. 1:** Radiografia anteroposterior e de perfil no pronto-socorro.

**Fig. 2:** Cortes coronais da tomografia computadorizada do joelho.

**Fig. 3:** Cortes axiais e sagitais da tomografia computadorizada do joelho.

Primeiramente, abordamos o platô tibial medial. Por uma incisão posteromedial, reduzimos a fratura de separação com auxílio de fluoroscopia e colocamos uma placa medial temporária com fios de Kirschner. Em seguida, realizamos um procedimento artroscópico de dois portais, com remoção de *debris* e irrigação. Então, procedemos à exploração da fratura do platô tibial em busca de lesões intra-articulares associadas. Satisfeitos com a redução do platô medial, fizemos a fixação permanente da placa. A seguir, passamos para a eminência tibial: como o desbridamento já havia sido feito e não existia encarceramento do menisco, colocamos a guia da broca Director ACUFEX (Smith and Nephew, Watford, Inglaterra, Reino Unido) do instrumental dos ligamentos cruzados na face anterior do leito da fratura da eminência e criamos um único túnel através da borda anterior da tíbia proximal. Depois, colocamos uma sutura ULTRATAPE (Smith and Nephew, Watford, Inglaterra, Reino Unido) pelos portais artroscópicos para transfixação das fibras do ligamento cruzado anterior o mais perto possível dos fragmentos ósseos inferiores ( Fig. 4 ); então, a sutura foi passada pelo túnel tibial para reduzir a fratura da eminência e fixada com um *endobutton* com o joelho em extensão total ( Fig. 5 ).

**Fig. 4:** Vista artroscópica da fita de transfixação do ligamento cruzado anterior e tração para baixo da fratura da eminência.

**Fig. 5:** Vistas fluoroscópicas anteroposterior e lateral intraoperatórias.

Após a cirurgia, a paciente usou uma órtese De Puy (imobilizante genérico 0° do joelho) sem carga por 15 dias. O programa de reabilitação começou imediatamente após o procedimento, em ambiente ambulatorial, com estimulação elétrica neuromuscular do quadríceps e dos isquiotibiais com o joelho em extensão completa. A etapa seguinte do programa de reabilitação compreendeu o fortalecimento isométrico dos adutores e abdutores do quadril, retirada gradativa da órtese e mobilização passiva do joelho com graus progressivos de flexão, tendo como objetivo principal a melhora da amplitude de movimento. Um mês após a cirurgia, a sustentação de peso sem dor foi permitida e o foco foi o fortalecimento muscular do quadríceps, dos isquiotibiais e do gastrocnêmio, começando com exercícios isométricos e progredindo por 6 semanas depois do procedimento para exercícios dinâmicos e pliométricos com flexão resistida em toda a amplitude de movimento e extensão resistida entre 30 e 90° de flexão. A extensão resistida entre 0 e 30° foi permitida 3 meses após a cirurgia. Exercícios proprioceptivos de cadeia cinética aberta foram iniciados às 6 semanas e progrediram a exercícios de cadeia cinética fechada.

Aos 3 meses, a paciente não referia dor, não usava muletas e apresentava bom alinhamento radiográfico ( Fig. 6 ), sustentação total de peso e amplitude de movimento do joelho de 0 a 90° ( Fig. 7 e 8 ).

**Fig. 6:** Radiografia anteroposterior e lateral no terceiro mês de acompanhamento.

**Fig. 7:** Flexão no terceiro mês pós-operatório.

**Fig. 8:** Extensão no terceiro mês pós-operatório.

Em 1 ano de acompanhamento, a paciente não relatou limitação em suas atividades de vida diária, com escore International Knee Documentation Committee (IKDC, na sigla em inglês) de 74,7, resultados negativos aos testes de Lachman e gaveta anterior e nenhum impedimento para atividades cotidianas.

## Discussão

A relevância da fratura da eminência tibial no contexto de uma fratura do platô tibial é pouco discutida na literatura atual; 5 sugere-se, porém, que 19,4% das fraturas do platô tibial estão associadas a uma fratura da eminência tibial, e que 84,3% das fraturas da coluna tibial em adultos coexistem com uma fratura do platô tibial. 2

A literatura traz alguns relatos sobre abordagens e resultados nesta combinação específica de lesões.

Di Caprio et al. 2 realizaram um estudo com 29 pacientes com fratura de platô e da eminência tibial e descreveram 2 técnicas artroscópicas, semelhantes à nossa, para abordagem dessas lesões, com bons resultados. Essas técnicas artroscópicas e de sutura *pull-out* têm como vantagens a simplicidade, o baixo índice de complicações intraoperatórias e tensão adequada do ligamento cruzado anterior. Uma particularidade deste estudo foi o tratamento de todas as fraturas do platô tibial com parafusos de fixação percutânea.

Huang et al. 1 também estudaram esta lesão combinada em uma análise retrospectiva com acompanhamento mínimo de 5 anos, usando técnica idêntica de 2 túneis. Embora seus resultados tenham sido comparáveis aos obtidos por Di Caprio et al., 2 os autores afirmaram que a técnica não interferiu na fixação da placa e do parafuso na fratura do platô tibial, assim como em nosso caso.

Lubowitz et al. 3 descreveram outra técnica equivalente para fixação da eminência tibial com suturas não absorvíveis, relatando como vantagens a eliminação do risco de cominuição do fragmento da fratura, da necessidade de retirada do material de síntese e do risco de lesão neurovascular posterior.

Em relação aos desfechos, Konda et al. 5 compararam pacientes com esta lesão combinada àqueles com fraturas isoladas do platô tibial e observaram menor velocidade de recuperação no primeiro grupo, mas resultados funcionais comparáveis em 1 ano de acompanhamento. Sapre et al. 11 também encontraram bons resultados após a redução anatômica com várias técnicas artroscópicas.

No presente artigo, descrevemos mais uma técnica cirúrgica combinada envolvendo artroscopia, sutura não absorvível e fixação com placa desta lesão combinada, com bom resultado em 1 ano de acompanhamento. Um programa de reabilitação oportuno e personalizado também é crucial para o resultado clínico. Estes dados corroboram os estudos supracitados de que esta é uma excelente abordagem cirúrgica única para uma lesão complexa do joelho, sem incompatibilidade entre o material usado no platô tibial e o método de fixação da eminência e com bons tempos de recuperação e altos índices de satisfação.
